# Short Term Virologic Efficacies of Telbivudine versus Entecavir against Hepatitis B-Related Hepatocellular Carcinoma

**DOI:** 10.1155/2015/181065

**Published:** 2015-03-24

**Authors:** Young Woon Kim, Jung Hyun Kwon, Eun Chung, Sung Won Lee, Jong-yul Lee, Jeong Won Jang, Kyu Won Chung, Soon Woo Nam

**Affiliations:** ^1^Catholic Liver Research Center, College of Medicine, The Catholic University of Korea, Seoul, Republic of Korea; ^2^Department of Internal Medicine, Incheon St. Mary's Hospital, Catholic University of Korea, Bupyeong 6-dong, Bupyeong-gu, Incheon 403-720, Republic of Korea; ^3^Department of Internal Medicine, Seoul St. Mary's Hospital, College of Medicine, The Catholic University of Korea, Seoul, Republic of Korea

## Abstract

Telbivudine has been reported to be more effective than lamivudine. However, because of the resistance rate to telbivudine (TLV), the current guidelines recommend entecavir (ETV) or tenofovir (TNV) as the first-line therapy for chronic hepatitis B. We investigated the short term virologic efficacy of TLV in comparison with ETV as the first-line agent of HBV suppression in HBV-related advanced HCC patients. A total of 86 consecutive patients with HBV-related HCC for whom antiviral treatment was initiated in Incheon St. Mary's Hospital between 2010 and 2013 were analyzed. Virologic responses were investigated on the 4th, 12th, and 24th weeks of the antiviral therapies. In patients with advanced TNM stage cancer (stage 3 or 4) and poor liver function (Child-Pugh class B or C), the virologic response rates at weeks 12 and 24 were 25% (1/4) and 42.8% (3/7) in the TLV group and 33.3% (1/3) and 33.3% (1/3) in the ETV group, respectively (*P* = 0.424, *P* = 0.800). The short term efficacy of TLV was similar to that of ETV. Since TLV is highly cost-effective, it should be considered as a first-line antiviral agent in patients with advanced HCC, poor liver function, and short life expectancies.

## 1. Introduction

Hepatocellular carcinoma (HCC) is common in Asia. Moreover, this disease is usually detected in its advanced stages and is thus associated with low survival rates and poor prognoses [1, 2]. Several factors can affect patient's survival and prognosis, including the tumor size, number of tumors, extent of vessel invasion, and presence of extrahepatic metastasis; however, the coexistence of underlying liver disease and/or impaired hepatic function is especially important in the context of HCC.

An association between chronic hepatitis B (CHB) and HCC has been demonstrated in several studies. In particular, 70–80% of all patients in Asia with HCC are also infected with hepatitis B virus (HBV), and a large number of patients worldwide with HCC are also simultaneously diagnosed with HBV infection. Moreover, HBV infection is also known to affect prognosis and treatment strategies. For example, HBV reactivation or exacerbation can complicate systemic chemotherapy, radiation therapy, or even surgical therapy procedures for patients with HBV-related HCC. Thus, effectively controlling HBV infection and maintaining functional hepatic reserve are extremely important in the management of patients with HBV-related HCC [3, 4].

Many antiviral treatments employing nucleoside analogues have been successfully developed over the past two decades. Lamivudine (LAM) was the first popular antiviral agent against CHB. LAM was effective, well tolerated, and inexpensive; however, the development of LAM-resistant HBV limited the application of this therapeutic. Telbivudine (TLV) has been reported to be more effective than LAM against HBV; moreover, significantly less resistance to TLV has been observed in patients with chronic hepatitis B compared with LAM [5]. Although the resistance rate to TLV has typically been lower than that to LAM, strains of TLV-resistant HBV have begun to emerge. Thus, the current guidelines recommend entecavir (ETV) or tenofovir (TNV) as the first-line therapy for CHB due to their excellent efficacies and low resistance rates; however, these two therapeutic approaches are much more expensive than LAM [6, 7]. However, if resistance is the main reason for avoiding treatment of patients with chronic hepatitis with TLV, TLV treatment can be reconsidered for patients with advanced HCC and only short term prospects for survival.

We hypothesized that TLV would be a suitable first-line treatment for patients with advanced HCC, since there is little chance that resistance to TLV will develop over a short survival time. Moreover, TLV is much less expensive than either ETV or TNV in South Korea and also in most other countries. Here, we compared the efficacies of TLV and ETV for treating antiviral-naïve patients with HBV-related advanced HCC, with the aim of determining whether TLV is an effective first-line antiviral agent.

## 2. Patients and Methods

### 2.1. Patients

**Figure 1:**
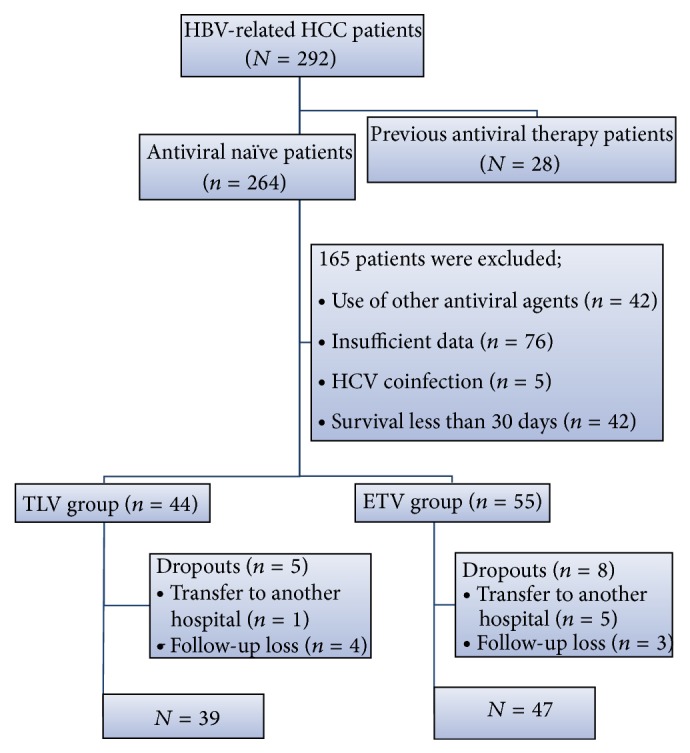
Flow diagram of study population selection. HBV, hepatitis B virus; HCC, hepatocellular carcinoma; TLV, telbivudine; and ETV, entecavir.

Between January 2010 and June 2013, a total of 292 patients with HBV-related HCC were diagnosed at Incheon St. Mary's Hospital, The Catholic University of Korea. Patients meeting the following criteria were excluded from the study: had previously undergone an antiviral therapy for CHB, had used an antiviral agent other than TLV or ETV, had a survival time of less than 30 days after enrollment, had insufficient data available, exhibited coinfection with hepatitis C or D virus, had been transferred to another hospital, or were lost during the follow-up period. After application of these criteria, a total of 86 consecutive patients were included in this study (Figure 1). Our study was in accordance with the ethical standards of our institution (Catholic Medical Center Human Research Protection Program). The ethics committee deemed that patient consent was not required, since only retrospective samples were used.

### 2.2. Study Design and Definitions

The primary endpoint was the comparison of the antiviral efficacies of TLV and ETV in antiviral therapy-naïve patients with HBV-related HCC. Hepatitis B e-antigen (HBeAg), antibodies to HBeAg (anti-HBe), and levels of HBV DNA were assessed at baseline and then again at weeks 4, 12, and 24. Biochemical data, including the concentration of alanine transaminase (ALT), the concentration of aspartate aminotransferase (AST), the amount of total bilirubin, the amount of albumin, the prothrombin time, the level of alpha-fetoprotein (AFP), and the platelet count were collected at baseline. Indication for antiviral treatment was based on the guidelines of the Korean Association for the Study of Liver Disease [8]. A virologic breakthrough (VB) was defined as a confirmed increase in the level of HBV DNA (>1 log_10_ IU/mL compared with the nadir of HBV DNA while the patient was receiving therapy). HBeAg seroconversion was defined as the loss of HBe Ag with the development of anti-HBe. HBV DNA suppression and HBV DNA nondetection were defined as <2000 IU/mL and 20 IU/mL of HBV DNA, respectively. Genotypic resistance (GR) was defined as the appearance of an HBV genomic mutation that conferred resistance to antiviral agents. A primary nonresponder to TLV and ETV was defined as a patient with a <2 log_10_ IU/mL reduction in the concentration of HBV DNA over 6 months.

### 2.3. Diagnosis of HCC and Staging System

Diagnosis of HCC was based on pathologic confirmation, the typical appearance of HCC on two different dynamic imaging examinations (computed tomography and magnetic resonance imaging), or on one dynamic technique if accompanied by an elevated level of serum a-fetoprotein (AFP; >400 ng/mL) [9]. Tumor staging was performed according to the TNM Classification of Malignant Tumors/International Union against Cancer (UICC) classification system, which is widely used in Korea [9].

### 2.4. Laboratory Assays

The levels of serologic markers, including hepatitis B surface antigen (HBsAg), hepatitis B e antigen (HBeAg), and antibodies to HBeAg, were measured with commercial immunoassays (Abbott Laboratories, Abbott Park). Serum HBV DNA viral loads were quantified using a COBAS Ampli-Prep-COBAS TaqMan assay (detection limit: 20 IU/mL; Roche Diagnostic, Branchburg, NJ). The HBV genotypes were determined by restriction fragment mass polymorphism analysis. Biochemical data, including ALT, AST, total bilirubin, albumin, prothrombin time, and platelet count values, were measured using a sequential multiple autoanalyzer (Hitachi, Tokyo, Japan).

### 2.5. Statistical Analysis

Data are expressed as means ± SD or medians (ranges). Continuous and categorical variables were compared between groups using the Mann-Whitney test; nonparametric continuous data were compared using Kruskall-Wallis ANOVA. The Kolmogorov-Smirnov test was applied to determine whether the sample data were likely to be derived from a normally distributed population. Student's *t*-test or ANOVA was used to compare normally distributed data, whereas the Wilcoxon signed rank test or the Mann-Whitney *U* test was used to compare nonparametric continuous data. *P* values < 0.05 were considered statistically significant. The Kaplan-Meier method was used to calculate survival rates and the log-rank test was used for univariate analysis. Statistical analyses were performed using the Statistical Package for Social Science (SPSS Inc., Chicago, IL, USA), version 18.0.

## 3. Results

### 3.1. Baseline Characteristics of Patients

A total of 86 consecutive antiviral-naïve patients with HBV-related HCC were enrolled in this study. After diagnosis of HCC, ETV (0.5 mg) treatment was initiated with 47 (54.6%) patients (ETV group), whereas TLV (600 mg) treatment was initiated with 39 (46.4%) patients (TLV group) (Figure 1). The baseline characteristics of the TLV and ETV groups are shown in Table 1. The levels of HBV DNA and HBe positivity did not differ significantly between the TLV and ETV groups (5.91 ± 0.96 log_10_ IU/mL* versus *5.92 ± 1.33 log_10_ IU/mL, *P* = 0.968; 21 (53.8%)* versus* 25 (53.1%), *P* = 0.952). However, the HCC stages, levels of alpha fetoprotein, and death rates were significantly different between the two groups (all *P* > 0.05). All patients (except 3 patients in the TLV group) received anticancer treatment, including hepatectomy, radiofrequency ablation, transarterial chemoembolization, and sorafenib.

### 3.2. Efficacy of Antiviral Therapy

The results of the TLV and ETV therapies are shown in Table 2. The level of HBV DNA was consistently reduced in both the TLV and ETV groups, after treatment with the appropriate antiviral agent was initiated, throughout the entire 24 weeks. On the 12th and 24th weeks, the levels of HBV DNA were higher in the TLV group compared with the ETV group; however, these differences were not significant (*P* = 0.253 and *P* = 0.348, resp.), even though the baseline levels of HBV DNA were similar in the two groups. Hepatitis B virus DNA levels <2,000 copies/mL were achieved in 81.8% and 87.5% of all patients at 24 weeks in the TLV and ETV groups, respectively. The nondetection rates for HBV DNA on the 12th and 24th weeks were 3 (21.4%) and 2 (18.1%) in the TLV group and 5 (18.5%) and 12 (37.5%) in the ETV group, respectively (*P* = 0.583 and *P* = 0.213). The rates of HBe antigen positivity were 21 (53.8%) in the TLV group and 25 (53.1%) in the ETV group. The rates of HBe antigen seroconversion were 2 (9.5%) in the TLV group and 2 (7.4%) in the ETV group (*P* = 0.119 and *P* = 0.248). No episodes of antiviral resistance or hepatitis flare ups were recorded during the observation period.

### 3.3. Efficacy of Antiviral Therapy in Patients with Advanced HCC

We analyzed the efficacies of TLV and ETV antiviral therapies in naïve patients with advanced HCC; otherwise, in previous paragraph, the analysis was done in the entire 86 HCC patients. The tumor, node, and metastases (TNM) stages III, IVa, and IVb and Child-Pugh grade B and grade C were included from entire populations, 86 patients. Disease was scored according to the modified Union for International Cancer Control (UICC) staging system and the Child-Pugh classification system. Out of the 25 patients with advanced HCC, 14 patients were in the TLV group and 11 patients were treated with ETV. The baseline characteristics of the TLV and ETV groups are shown in Table 3. There were differences in the demographic and laboratory characteristics of the two groups with sex (*P* = 0.149), CP class (*P* = 0.038), platelet count (*P* = 0.04), and the level of HBV DNA (log_10_ copies/mL) (*P* = 0.038). The significant differences in death rate and tumor stage (TNM stage), which were observed in an analysis of the entire population of patients with HBV-related HCC, were not observed when we restricted our analysis to patients with advanced HCC. The levels of HBV DNA (log_10_ copies/mL) were consistently reduced over the entire 24 weeks in both the TLV and the ETV groups after treatment with the appropriate antiviral agent had been initiated (Table 4). Similarly, the rates of HBV DNA suppression and nondetection were similar in TLV-treated and ETV-treated patients at 4, 12, and 24 weeks.

### 3.4. Survival Analysis and Causes of Death

**Figure 2:**
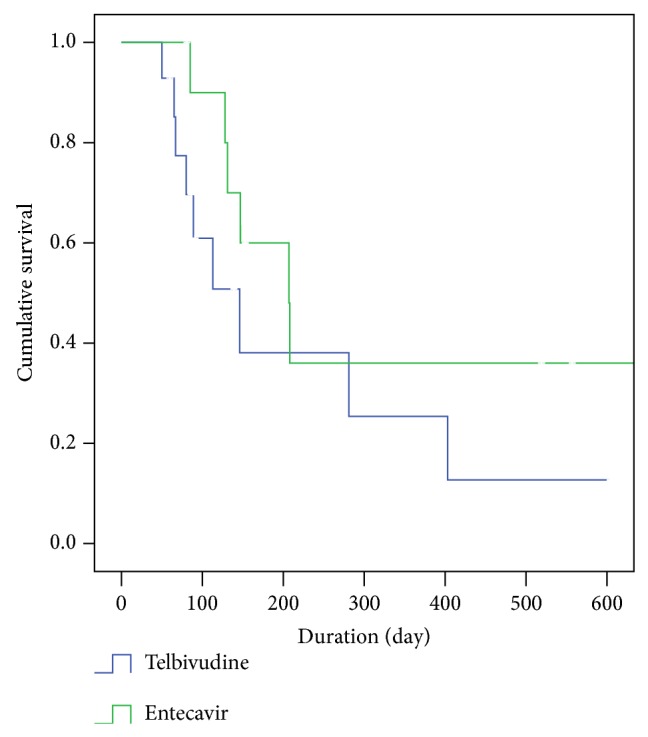
The Kaplan-Meier survival analysis of patients with HBV-related advanced HCC.

Survival analysis was performed to determine whether the first-line antiviral agent influenced the overall survival of patients with HBV-related advanced HCC. The median survival times were 146 (range: 152.5) and 207 (range: 163.3) days in the TLV and ETV groups, respectively. The specific antiviral agent used (TLV or ETV) did not influence patient's overall survival (log-rank test, *P* = 0.190) (Figure 2). During the entire study period, 9 patients in the TLV group and 6 patients in the ETV group died. Of these patients, 6 (66%) patients in the TLV group and 3 (50%) patients in the ETV group died of HCC progression. No patient in either group died of liver failure due to a hepatitis B flare up.

## 4. Discussion

Generally, antiviral treatment in patients with HBV-related HCC is a component of care that is as important to manage as the treatment of the HCC itself. The current treatments for HBV-related HCC, such as surgical resection, radiation therapy, and systemic chemotherapy, have resulted in life-threatening reactivation of HBV and exacerbation of hepatitis [10–12]. Nucleotide analog treatments can prevent the progression of liver failure and the development of HCC, thereby increasing the survival rates of patients with HCC by helping to improve their liver function [13, 14]. Jang et al. showed that the level of HBV DNA (>10^4^ copies/mL) and the treatment option were both independent predictors of HBV reactivation during HCC treatment; moreover, the reactivated disease became more severe as the treatment intensity increased [15]. Hence, antiviral treatments in patients with CHB-related HCC can influence the optimal HCC treatment and potentially can lead to better prognoses. Indeed, some case studies of LAM treatment of patients with HCC have demonstrated successful treatment of the tumors [16, 17].

TLV has been shown to be an effective antiviral therapy agent, with an efficacy superior to that of LAM over a two-year therapy course in patients with CHB; moreover, TLV has been shown to have excellent safety and tolerability profiles [5]. One study of previously untreated HBeAg-positive HBV infections found no statistically significant differences in either the effectiveness or the tolerability of TLV compared with ETV after 24 weeks of treatment [18]. However, the rates of viral breakthrough and emergence of genotypic resistance in the TLV-treated group were significantly different depending on HBeAg positivity, with rates of 5% in the first year and 25.1% in the first two years in HBeAg-positive patients compared with rates of 2.2% in the first year and 10.8% in the first two years in HBeAg-negative patients [5]. However, these rates are still significantly lower than those of LAM, which can exhibit resistance rates as high as 70% of all cases after 5 years [5, 19]. The rate of genotypic ETV resistance in nucleoside-naïve patients has been shown to be 1.2% over a 5-year course of therapy [20]. In addition, the absence of TNV resistance has been shown to be maintained for up to 3 years [21, 22]. The high rates of TLV resistance have deterred this antiviral from being used as a first-line therapeutic agent in patients with CHB. ETV and TNV, both of which exhibit high genetic barriers to the development of resistance, are better first-line antiviral therapies for patients with CHB [8]. However, since there is little opportunity for TLV resistance to develop in patients with advanced HCC due to their relatively short survival times, TLV can be chosen as the first-line antiviral agent in these patients. Also, TLV is much less expensive than both ETV and TNV, which is an important issue in developing countries.

To the best of our knowledge, no studies have examined the efficacy of TLV in patients with CHB-related HCC. In the present study, the antiviral efficacy of TLV in HCC treatment was fairly good, and the development of antiviral resistance to TLV was low. Moreover, these metrics did not differ significantly between the TLV and ETV groups. In addition, the overall survival rates of TLV-treated and ETV-treated patients with advanced TNM stage disease and poor liver function were not different (median 146* versus* 207 days; log-rank test, *P* = 0.190). Thus, we conclude that TLV is not inferior to ETV as a first-line agent for patients with HBV-related advanced HCC. However, patients predicted to have longer survival times, such as those with early or intermediate stage HCC, should be treated with ETV or TNV as a first-line antiviral agent, due to the potential emergence of TLV resistance over time.

This study did have some limitations. For instance, TLV treatment was initiated mainly in patients with advanced stage HCC and poor liver function, whereas ETV treatment was initiated regardless of the HCC stage or degree of liver function. This is basically a retrospective study, and selection bias of the patients cannot be avoided. Table 1 showed significant difference in several baseline characteristics in an analysis of the entire population of patients with HBV-related HCC. To fix the selection bias, the patients were enrolled with consecutive manner, and we subanalysed patients with advanced stage HCC and poor liver function (Tables 3 and 4). Other limitations include a relatively small number of patients with advanced HBV-related HCC. However, no differences were observed between the entire population of patients with HCC and the subset of patients with advanced HCC regarding antiviral efficacy, drug resistance, and amount of HBV flare ups in both the TLV group and the ETV group.

## 5. Conclusion

The data presented here indicate that TLV is an appropriate first-line antiviral agent for treating patients with HBV-related advanced HCC. Moreover, TLV is not inferior to ETV, which has a high genetic barrier to resistance and a high cost.

## Figures and Tables

**Table 1:** Baseline demographics and clinical characteristics.

	TLV (*n* = 39)	ETV (*n* = 47)	*P* value
Age, years	56.1 ± 9.5	60.5 ± 8.3	0.024
Male	31/8 (79%)	39/8 (76%)	0.683
CP class (A/B/C)	23/10/6	31/15/1	0.148
ALT, IU/L	89.6 ± 92.8	66.5 ± 134.3	0.365
Prothrombin time, INR	1.25 ± 0.22	1.22 ± 0.12	0.402
Albumin, g/dL	3.19 ± 0.61	3.41 ± 0.57	0.098
Total bilirubin, mg/dL	2.14 ± 1.93	1.36 ± 0.70	0.012
Platelet count, 10^3^/mL	175.5 ± 80.2	120.7 ± 64.4	0.001
Median alpha-fetoprotein (range), ng/mL	3859.5 (985558.3)	69.43 (33776.3)	0.003
HBV DNA, log_10_⁡ IU/mL	5.91 ± 0.96	5.92 ± 1.33	0.968
HBeAg positivity	21 (53.8%)	25 (53.1%)	0.952
Tumor stage, TNM stages I/II/III/IVa/IVb	0/3/7/15/14	8/15/9/8/7	0.000
Death	27/39 (69.2%)	16/47 (34%)	0.001
Treatment modality			
RFA	0	2 (5.1%)	
Hepatectomy	2 (4.2%)	12 (30.7%)	
TACE	32 (82%)	33 (70.2%)	
Sorafenib	2 (4.2%)	0	
RT	0	0	
Conservative care	3 (6.3%)	0	

Variables are expressed as means ± standard deviation (SD), medians (ranges), or *n*/*N* (%). TLV, telbivudine; ETV, entecavir; CP, Child-Pugh; ALT, alanine transaminase; INR, international normalized ratio; HBeAg, hepatitis B e antigen; TNM, tumor-node-metastasis; RFA, radiofrequency ablation; TACE, transarterial chemoembolization; and RT, radiation therapy.

**Table 2:** Outcomes of antiviral therapy, as indicated by the mean reduction in serum DNA, HBV DNA suppression, undetectable HBV-DNA, and HBe seroconversion.

	TLV (39)	ETV (47)	*P* value
HBV DNA (log_10_⁡ IU/mL)			
4th week	3.32 ± 0.80	3.64 ± 1.06	0.228
12th week	2.94 ± 0.96	2.54 ± 1.02	0.253
24th week	2.24 ± 1.39	1.9 ± 0.82	0.348
HBV DNA suppression, (below 2000 IU/mL)			
4th week	13/31 (41.9%)	15/32 (46.8%)	0.444
12th week	11/14 (78.6%)	22/27 (81.5%)	0.565
24th week	9/11 (81.8%)	28/32 (87.5%)	0.488
Nondetectable HBV DNA (below 20 IU/mL)			
4th week	0/31	0/32	
12th week	3/14 (21.4%)	5/27 (18.5%)	0.583
24th week	2/11 (18.1%)	12/32 (37.5%)	0.213
HBeAg seroconversion			
4th week	2/21 (9.5%)	0/25	0.119
12th week	0/14	2/27 (7.4%)	0.248
24th week	0/11	0/32	
Antiviral resistance	0	0	
Hepatitis flare up	0	0	

Variables are expressed as mean ± standard deviation (SD) or *n*/*N* (%). Hepatitis flare up was defined as the elevation of alanine aminotransferase (ALT) level to more than 10 times the upper normal limit. TLV, telbivudine; ETV, entecavir; and HBeAg, hepatitis B e antigen.

**Table 3:** Baseline demographics and clinical characteristics of patients with advanced HBV-related HCC.

	TLV (*n* = 14)	ETV (*n* = 11)	*P* value
Age, years	55.8 ± 8.5	60.4 ± 6.3	0.149
Male	9	11	0.038
CP class, B/C	9 (64.2%)/5 (35.7%)	11/0	0.038
ALT, IU/L	94.5 ± 108.8	122.0 ± 267.6	0.728
Prothrombin time, INR	1.35 ± 0.25	1.3 ± 0.12	0.524
Albumin, g/dL	2.82 ± 0.44	3.00 ± 0.47	0.362
Total bilirubin, mg/dL	3.07 ± 2.45	1.95 ± 0.61	0.156
Platelet count, 10^3^/mL	159.1 ± 90.9	95.4 ± 36.3	0.040
Median alpha fetoprotein, range, ng/mL	37707.1 (715902.5)	298.9 (27346)	0.053
HBV DNA concentration, log_10_ IU/mL	6.40 ± 0.71	5.45 ± 1.40	0.038
HBeAg positivity	9 (64.2%)	5 (45.6%)	0.296
Tumor stage TNM stages III/IVa/IVb	3/5/6	5/4/2	0.319
Mean observation time (days)	162.4 ± 160.5	265.9 ± 219.7	0.622
Death	9 (64.2%)	6 (54.5%)	0.466

Variables are expressed as means ± standard deviation (SD), medians (ranges), or *n*/*N* (%). TLV, telbivudine; ETV, entecavir; CP, Child-Pugh; ALT, alanine transaminase; INR, international normalized ratio; HBeAg, hepatitis B e antigen; and TNM, tumor-node-metastasis.

**Table 4:** Outcomes of antiviral therapy in patients with HBV-related advanced HCC, as indicated by the mean reduction in serum DNA, HBV DNA suppression, undetectable HBV-DNA, and HBe seroconversion.

	TLV (14)	ETV (11)	*P* value
HBV DNA concentration, mean (SD), (log_10_⁡ IU/mL)			
4th week	3.38 ± 0.58	3.72 ± 0.94	0.328
12th week	3.20 ± 1.32	2.15 ± 0.94	0.178
24th week	1.76 ± 1.54	1.39 ± 1.04	0.697
HBV DNA suppression (below 2000 IU/mL)			
4th week	4/12 (33.3%)	4/11 (36.3%)	0.611
12th week	3/4 (75%)	6/7 (85.7%)	0.400
24th week	3/3 (100%)	2/3 (66.6%)	0.500
Nondetectable HBV DNA (below 20 IU/mL)			
4th week	0/12	0/9	
12th week	1/4 (25%)	3/7 (42.8%)	0.424
24th week	1/3 (33.3%)	1/3 (33.3%)	0.800
HBe seroconversion			
4th week	1/12 (8.3%)	0/9	0.560
12th week	0/4	0/7	
24th week	0/3	0/3	
Antiviral resistance	0	0	
Hepatitis flare up	0	0	

Variables are expressed as mean ± standard deviation (SD) or *n*/*N* (%). Hepatitis flare up was defined as the elevation of alanine aminotransferase (ALT) level to more than 10 times the upper normal limit. TLV, telbivudine; ETV, entecavir; and HBeAg, hepatitis B e antigen.
